# Identification of Lupus Erythematosus Cells and Ragocytes in Peripheral Blood from a Dog with Systemic Lupus Erythematosus

**DOI:** 10.1111/vcp.70087

**Published:** 2026-05-07

**Authors:** Samantha L. Braman, Jeremy P. Bessett, Katie L. Anderson, Kristen R. Friedrichs

**Affiliations:** ^1^ Department of Medical Sciences, School of Veterinary Medicine University of Wisconsin‐Madison Madison Wisconsin USA; ^2^ Department of Pathobiological Sciences, School of Veterinary Medicine University of Wisconsin‐Madison Madison Wisconsin USA

**Keywords:** lameness, lupus, neutrophil, synovial

## Abstract

A 3‐year‐old female‐intact German Shepherd presented for the evaluation of progressive shifting leg lameness, carpal swelling, and weight loss with a poor appetite since adoption about three months prior. Routine blood smear evaluation, lymph node, and synovial fluid cytologies all revealed rare neutrophils with cytoplasmic inclusions consistent with “Lupus Erythematosus (LE) cells” and “ragocytes”. The combination of clinical signs, cytologic findings, and high antinuclear antibody (ANA) titer in the absence of identifiable triggers were consistent with a diagnosis of systemic lupus erythematosus (SLE).

## 1 Case Report

A 3‐year‐old intact female German Shepherd presented to University of Wisconsin Veterinary Care (UWVC) for evaluation of progressive shifting leg lameness, carpal swelling, weight loss, and hyporexia since adoption about 3 months prior. The dog's history prior to adoption was unknown. The clinical signs worsened despite therapy with doxycycline, grapiprant, and cyclobenzaprine. Antibody testing for *Ehrlichia canis, Ehrlichia ewingii, Anaplasma phagocytophilum, Anaplasma platys, and Borrelia burgdorferi* and antigen testing for *Dirofilaria immitis* were negative (SNAP 4Dx, IDEXX). Serum biochemistry profile findings included increased liver enzymes (ALP > 915 U/L [reference interval {RI}, 20–150], ALT > 708 U/L [RI, 10–118]). A complete blood count was unremarkable. One week prior to referral, the dog was prescribed metronidazole, amoxicillin, and capromorelin. Despite a reduction in the carpal swelling, the dog's lameness progressed.

On physical exam at UWVC, the dog had a body condition score of 3/9 with a 6.4 kg decrease in weight over 3 months' time, significant forelimb lameness (right > left) with a stilted gait, bilateral carpal effusion (right > left), and multiple ulcer‐like lesions on the lips and tongue. Complete blood count, serum biochemistry profile, urinalysis, and urine protein creatinine (UPC) ratio were performed, and results are shown in Table 1.

**TABLE 1:** UWVC hematologic, serum biochemical, and urinalysis data.

Analyte	Initial presentation	Recheck (1 year)	Reference interval	Units
RBC	4.74	6.40	5.6–8.4	× 10^6^/uL
HGB	11.4	15.5	14–21	g/dL
HCT	34	46	39–57	%
PCV	33	44	40–59	%
MCV	71	72	61–73	fL
MCH	24	24	22–26	pg
MCHC	34	34	34–38	g/dL
Reticulocytes	0.136	0.030	0.013–0.102	× 10^6^/uL
WBC	13.4	13.8	5–14	× 10^3^/uL
Segmented neutrophils	9.2	12.5	2.6–10	× 10^3^/uL
Lymphocytes	2.8	0.7	0.7–4.3	× 10^3^/uL
Monocytes	0.7	0.5	0.1–0.9	× 10^3^/uL
Eosinophils	0.5	0.1	0.1–1.7	× 10^3^/uL
nRBC	0.1	0		× 10^3^/uL
Platelets	525	270	175–500	× 10^3^/uL
WBC toxic change	None	None		
BUN	22	27	7–32	mg/dL
Creatinine	0.9	0.8	0.5–1.5	mg/dL
Total protein	7.6	5.9	4.8–6.9	g/dL
Albumin	3.1	3.5	2.3–3.9	g/dL
Globulin	4.5	2.4	2.2–3.5	g/dL
CK	663	106	22–491	U/L
AST	63	27	21–53	U/L
ALT	51	208	14–87	U/L
ALP	343	187	20–157	U/L
GGT	< 10	13	5–16	U/L
Total bilirubin	0.5	0.2	0.1–0.8	mg/dL
Cholesterol	281	245	149–319	mg/dL
USG	1.021	1.031		
Urine protein (dipstick)	1+	Not obtainable^a^		
Urine protein (SSA)	Trace	Not obtainable^a^		
Urine Heme	Trace	Negative		
Urine pH	6.5	8		
Urine sediment RBC	None Seen	None seen		
Urine protein: creatinine	0.52	Not performed		

^a^ Result not obtainable due to high urine pH.

A blood film was prepared and stained routinely with a methanolic Modified Wright‐Giemsa stain (Aerospray Hematology Pro, Wescore Inc.). Upon examination, there were a total of three neutrophils throughout the blood film with several variably sized small, eosinophilic to lavender, round cytoplasmic inclusions consistent with ragocytes (Figure 1), and a single neutrophil with a single, large, round, eosinophilic cytoplasmic inclusion consistent with a lupus erythematosus (LE) cell (Figure 2).

**FIGURE 1:**
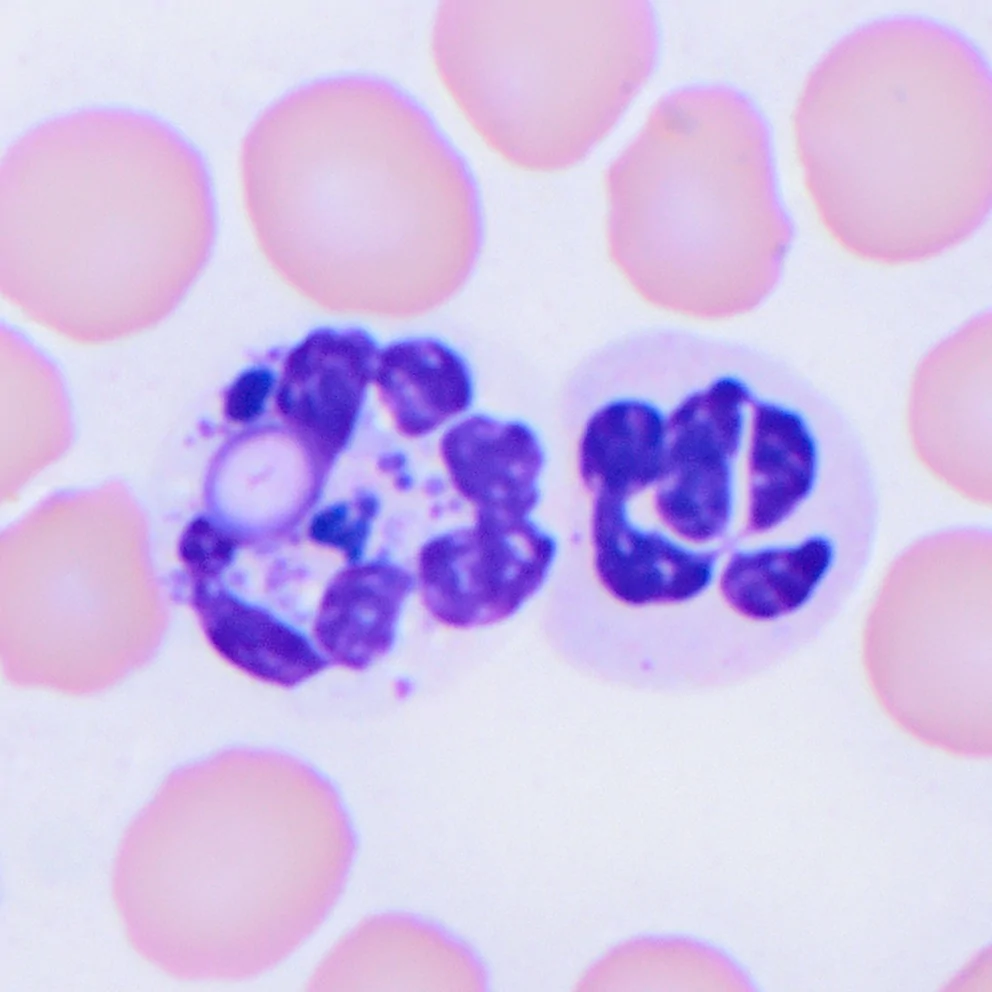
Peripheral blood film. Normal neutrophil (right) and neutrophil (left) containing several variably sized small, eosinophilic to lavender, round cytoplasmic inclusions consistent with a ragocyte. Modified Wright‐Giemsa stain (methanolic); Original magnification, ×1000.

**FIGURE 2:**
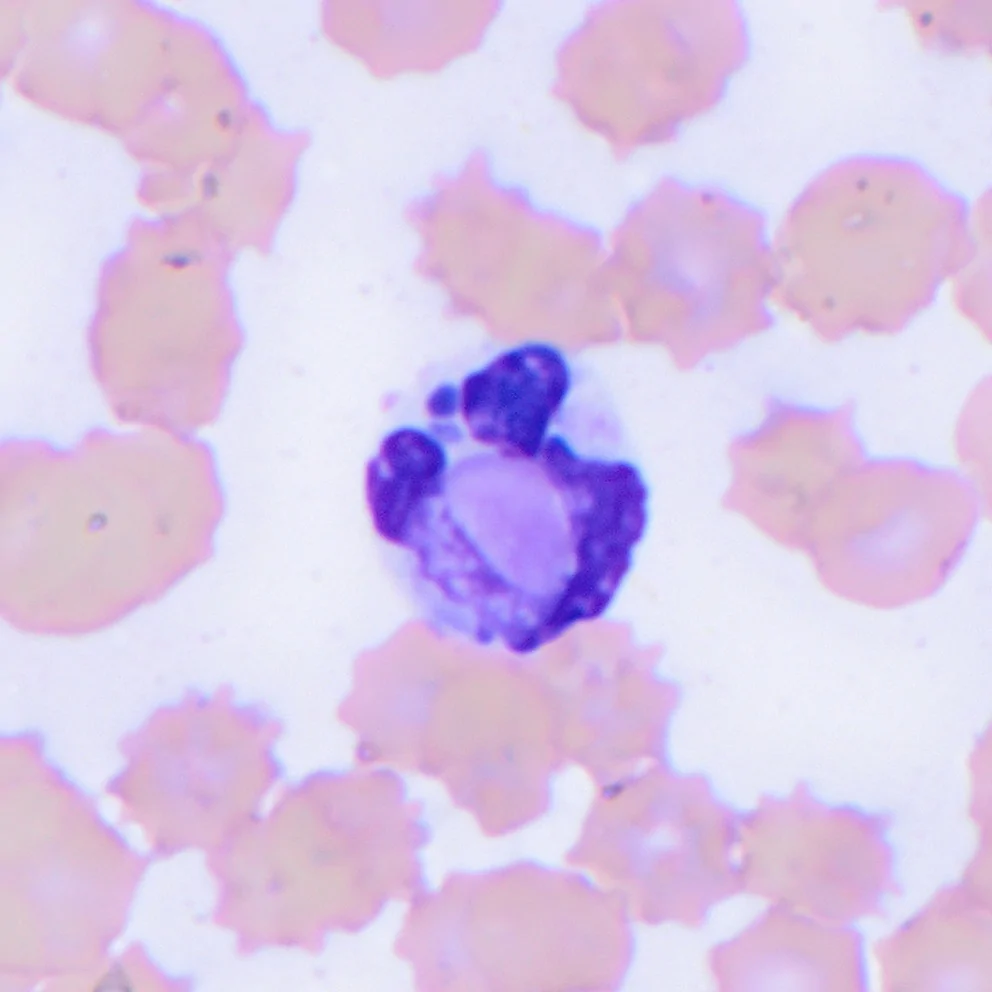
Peripheral blood film. Neutrophil with a single, large, round, eosinophilic cytoplasmic inclusion that displaces the nucleus, consistent with an LE cell. Modified Wright‐Giemsa stain (methanolic); Original magnification, ×1000.

Bilateral carpal radiographic findings included mild intracapsular soft tissue swelling surrounding the right carpus; the left carpus imaging was normal. Abdominal ultrasonographic findings included bilateral medial iliac lymphadenopathy (lymph node height and length: right 1.0 × 2.8 cm; left 1.17 × 3.26 cm), a liver that was hypoechoic to the spleen with a diffusely coarse echotexture, a moderate amount of heteroechoic, granular material within the gallbladder, and irregular cortical margins of both kidneys.

Ultrasound‐guided fine‐needle aspiration (FNA) of the liver and the right medial iliac lymph node was performed with specimens submitted to the UWVC clinical pathology laboratory. Slides were stained as described above and examined. The medial iliac lymph node was highly cellular and consisted of a heterogeneous lymphocyte population with increases in large lymphocytes and plasma cells. In some areas, there were variably sized aggregates of macrophages mixed with moderate numbers of neutrophils. A rare neutrophil contained a single, round, smooth, light purple inclusion that was encircled by and displaced the nucleus. Lymph node findings were consistent with reactive lymphoid hyperplasia, pyogranulomatous inflammation, and rare LE cells. The liver was highly cellular and consisted of monolayer sheets of hepatocytes. Hepatocyte cytoplasm was characterized by moderate amorphous cytoplasmic clearing, consistent with non‐lipid vacuolar hepatopathy.

Arthrocentesis was performed bilaterally on carpi and stifles. Direct smears were prepared and submitted to the UWVC clinical pathology laboratory. Slides were stained as described above and examined. Total nucleated cell counts could not be performed due to insufficient volume; however, the total nucleated cell concentrations were estimated to be moderately to markedly increased in all four sampled joints. Nondegenerate neutrophils were the dominant cell population (81%–97%) with fewer mononuclear cells (small lymphocytes, macrophages, and synovial lining cells). Occasionally, large mononuclear cells contained hemosiderin or erythrocytes, consistent with low‐level hemorrhage. Neutrophils frequently contained several variably sized small, eosinophilic to lavender, round cytoplasmic inclusions (Figure 3). Rarely to occasionally, neutrophils contained a single, medium to large, round, smooth, variably purple inclusion that was often encircled by and displaced the nucleus (Figure 3 Inset). Overall, the synovial fluid samples were consistent with moderate to marked neutrophilic inflammation with frequent ragocytes and occasional LE cells.

**FIGURE 3:**
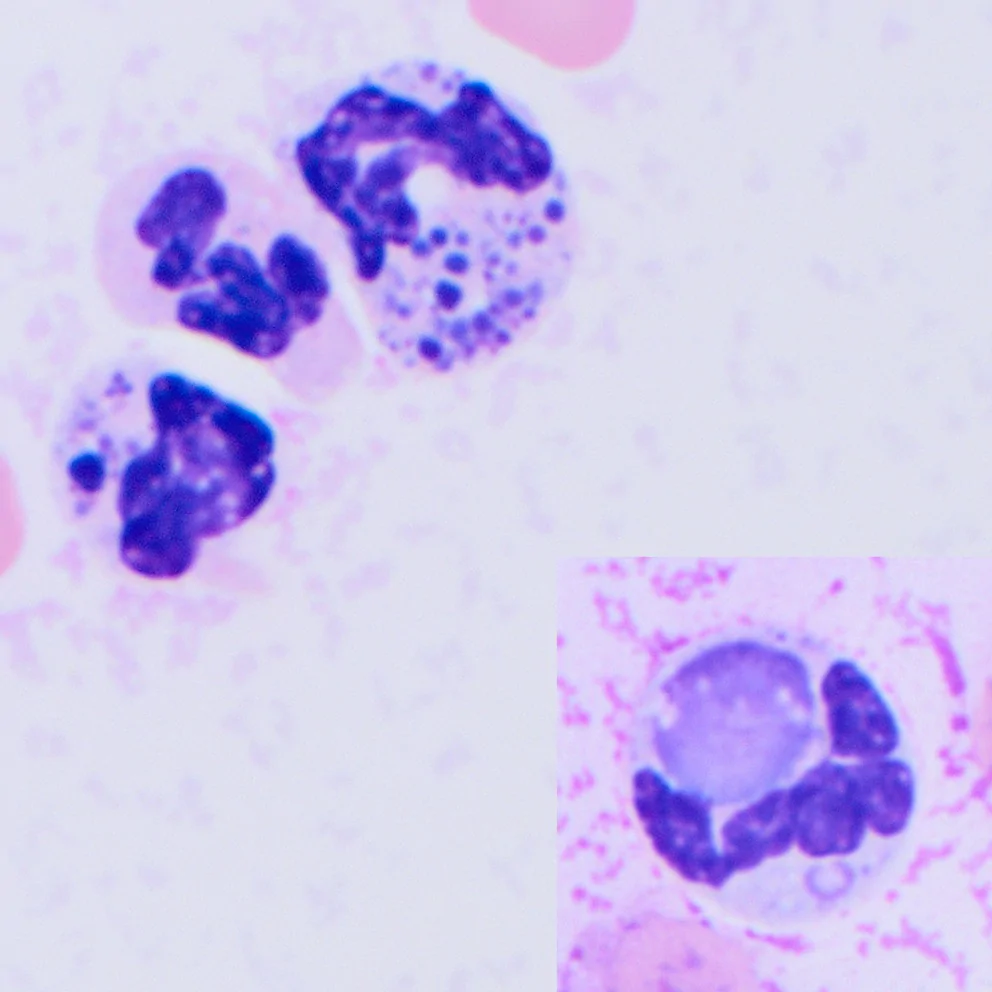
Joint Fluid. Two neutrophils contain many variably small, lavender to basophilic, round to irregular cytoplasmic inclusions (Ragocytes). One neutrophil contains no inclusions. Inset: One neutrophil contains a single, large, smooth, round, lavender cytoplasmic inclusion surrounded by a nucleus (LE cell). Modified Wright‐Giemsa stain (methanolic); Original magnification, ×1000.

Additional tick‐borne antibody testing (immunofluorescence assay for *Rickettsia rickettsii, Bartonella* sp., *Ehrlichia canis*, and *Babesia canis*, and ELISA for *Babesia gibsoni*) and PCR testing (*Anaplasma* genus, *Anaplasma platys, Anaplasma phagocytophilum, Babesia* genus, Apicomplexa, *Bartonella* genus, *Ehrlichia* genus, hemotropic *Mycoplasma* genus, and *Rickettsia* genus) were submitted to North Carolina State University. Serum was submitted for *Toxoplasma* sp. antibody titers (IgG and IgM) to Colorado State University. All tests for infectious agents were reported to be negative. Serum was submitted for an antinuclear antibody (ANA) titer to Cornell University and was positive at 1:5120 (negative cut‐off ≤ 1:80). Altogether, the physical exam findings (joint effusion, oral ulcer‐like lesions), cytologic findings, lack of an identifiable infectious process, and ANA titer were consistent with systemic lupus erythematosus (SLE).

Immunosuppression is the treatment of choice for SLE [1]. In this patient, mycophenolate was prescribed at the time of discharge prior to receiving the positive ANA titer. Due to concern for gastrointestinal bleeding based on the regenerative anemia and thrombocytosis, the dog was started on omeprazole and sucralfate, and prednisone was started 10 days after discharge with continuation of omeprazole and sucralfate therapy.

Approximately 1 year after the initial visit, the patient presented for a recheck examination. At this time, the patient no longer had signs of joint pain and mucocutaneous ulcers had resolved, having remained on mycophenolate and prednisone without attempted tapering. However, recent onset hindlimb weakness and ataxia were noted; neurologic examination was consistent with L6‐S1 myelopathy and possibly T3‐L3 myelopathy. Repeated CBC, serum biochemistry profile, and urinalysis were performed (Table 1). No ragocytes or LE cells were seen on a blood film review at this follow‐up visit. Arthrocentesis was not repeated in this patient, and further diagnostics to identify the cause of myelopathy were declined. No further follow‐up is available.

## 2 Discussion

SLE is a rare autoimmune disease characterized by multisystemic inflammation, with common clinical manifestations including pyrexia, polyarthritis, anemia, thrombocytopenia, and glomerulonephritis [2]. SLE is described in humans as a heterogeneous disease with variable manifestations affecting many organ systems. Cellular injury in target organs is shown to be caused by autoantibodies directed against DNA and chromosomal proteins (e.g., anti‐dsDNA, anti‐RO/SSA, anti‐LA/SSB, etc.) [2]. The cause and pathogenesis of SLE in animals is poorly understood and may be similar to that in people [1]. Certain infections, genetic predispositions, UV light exposure, and particular drugs have been associated with the development of SLE or SLE‐like diseases in people; however autoantibodies can be made in the absence of an identifiable trigger [2].

Definitive diagnosis of SLE in both human and animal patients is challenging. In people, diagnosis of SLE is based on guidelines from the American College of Rheumatology (ACR), which includes 11 criteria [3]. These criteria have been modified for SLE diagnosis in animals, which requires at least two manifestations of multisystemic autoimmune disease, including mucocutaneous lesions, nonerosive and nonseptic arthritis, glomerulonephritis, hemolytic anemia, thrombocytopenia, leukopenia, myositis, serositis, neurologic disorders, antiphospholipid antibodies along with a positive ANA titer in the absence of possible triggers for these findings [1]. This patient had met at least three criteria, including mucocutaneous lesions, nonerosive and nonseptic arthritis, as well as a positive ANA. Although the patient's UPC ratio was increased, and early glomerular proteinuria is possible, this cannot be determined as it was mild and could have been influenced by her systemic disease and inflammation [4]. The increased CK and AST may represent myositis, however biopsies were not taken to definitively determine this.

Although a positive ANA titer is often associated with SLE, it lacks specificity and can be present in several other non‐immune‐mediated diseases, including infectious (e.g., tick‐borne illnesses) or chronic inflammatory processes [1]. Although uncommonly identified, the presence of LE cells within synovial fluid is considered to be highly suggestive of SLE [5]. LE cells are neutrophils that contain aggregates of opsonized nuclear material [5]. Cytologically, these appear as smooth, round, cytoplasmic inclusions of varying sizes and coloration, which can be encircled by and displace the neutrophil nucleus [5]. Similarly, ragocytes are neutrophils that contain phagocytosed immune complexes or nuclear remnants, which can also be found in patients with SLE or other primary or secondary immune‐mediated inflammatory conditions [6].

The Feulgen staining method was developed in the 1920s and is believed to specifically stain DNA [7]. This stain has been utilized in previous case reports to provide additional evidence in support of observed inclusions consisting of nuclear material/DNA, which would be consistent with an LE cell [6]. However, Feulgen staining has been shown to be variable in LE cells formed in vivo due to the degradation of nuclear material within phagocytic cells [8]. Feulgen staining was not performed to confirm that the inclusions observed in this case were consistent with the material of ragocytes and/or LE cells; however, they appear morphologically consistent with what has previously been described for these cells [5, 6, 8]. The mucocutaneous lesions were not biopsied, and the mild regenerative anemia and mild increase in the UPC ratio were not further pursued diagnostically.

To our knowledge, this is the first reported case in veterinary medicine identifying LE cells on a peripheral blood film. Although an LE cell was also identified within the patient's lymph node sample, it cannot definitively be determined if this cell was from a peripheral blood contribution or truly formed within the lymph node tissue. This case highlights the utility of LE cell identification to aid the challenging diagnosis of SLE in veterinary patients.

## Conflicts of Interest

The authors declare no conflicts of interest.
